# Retraction: Synthesis of Al-based metal-organic framework in water with caffeic acid ligand and NaOH as linker sources with highly efficient anticancer treatment

**DOI:** 10.3389/fchem.2026.1959068

**Published:** 2026-08-18

**Authors:** Frontiers Editorial Office

The journal retracts the 30 November 2021 article cited above.

Following publication, concerns were raised about the citations and the integrity of the images in the published figures. The authors failed to provide a satisfactory explanation during the investigation, which was conducted in accordance with Frontiers’ policies.

This retraction was approved by the Chief Executive Editor of Frontiers. The authors received a communication regarding the retraction and had a chance to respond. This communication has been recorded by the publisher. The first author, Malihe Zeraati, does not agree to this retraction.

Frontiers would like to thank the users on PubPeer for bringing the published article to our attention.

